# Methods of Quantitative Assessment of the Response of Dilated Skin Blood Vessels to High-Energy Light Treatments

**DOI:** 10.3390/jcm13247547

**Published:** 2024-12-11

**Authors:** Anna Deda, Aleksandra Lipka-Trawińska, Barbara Błońska-Fajfrowska, Wiktoria Odrzywołek, Agata Lebiedowska, Magdalena Hartman-Petrycka, Dominika Wcisło-Dziadecka, Sławomir Wilczyński

**Affiliations:** 1 Department of Practical Cosmetology and Skin Diagnostics, Faculty of Pharmaceutical Sciences in Sosnowiec, Medical University of Silesia in Katowice, 10 Jednosci Street, 41-208 Sosnowiec, Poland; adeda@sum.edu.pl (A.D.); ddziadecka@sum.edu.pl (D.W.-D.); 2 Department of Basic Biomedical Science, Faculty of Pharmaceutical Sciences in Sosnowiec, Medical University of Silesia in Katowice, 10 Jednosci Street, 41-208 Sosnowiec, Poland; altrawinska@gmail.com (A.L.-T.); bbf@sum.edu.pl (B.B.-F.); wiktoria.odrzywolek@sum.edu.pl (W.O.); mhartman@sum.edu.pl (M.H.-P.); swilczynski@sum.edu.pl (S.W.)

**Keywords:** IPL, vascular lesions therapy, mexametric measurement, hemispheric directional reflectance, hyperspectral imaging

## Abstract

**Background:** The techniques of choice used in the treatment of extensive vascular lesions of the face are methods based on high-energy light sources, such as lasers and IPL (intense pulsed light). The techniques commonly employed to detect blood vessel abnormalities in skin primarily rely on semi-quantitative or qualitative scales. **Methods:** The study was conducted on a group of 38 volunteers; a series of three treatments was performed using an IPL source (Lumecca, Inmode, Israel). The vessels’ response to the high-energy light was verified using the following quantitative methods: mexametric measurements, hyperspectral imaging, and directional reflectance measurements. **Results:** In the mexameter measurement, statistically non-significant differences were obtained in the hemoglobin content in the skin condition prior to and following multiple intense pulsed light sessions. Studies performed using a hyperspectral camera showed that at a wavelength of 580 nm, the increase in skin reflectance after the treatment was statistically significant. Total directional reflectance measurements showed that at wavelengths of 400–540 nm and 480–600 nm, following the IPL treatment, the skin reflectance increased statistically significantly. Implementing three consecutive intense pulsed light procedures appears adequate to obtain a clinically satisfactory reduction in vascular changes in the facial skin. Mexametric measurements do not enable the assessment of the reaction of blood vessels to IPL. **Conclusions:** Hyperspectral imaging is an effective method for the quantitative assessment of skin vascular lesions. The best results in the assessment of vascular lesions using hyperspectral imaging are obtained at wavelengths of 420 nm and 580 nm. The hemispheric directional reflectance method allows for a quick, accurate, and repeatable assessment of vascular skin changes.

## 1. Introduction

The perception of skin color by the human eye depends on the absorption, scattering, or reflection of light in individual layers of the skin. Many factors influence a skin color, such as the content of skin chromophores, blood perfusion, stratum corneum thickness, and lichenification. The main chromophores in the skin that are responsible for absorbing light are melanin, hemoglobin, and oxyhemoglobin. Skin color is affected not only by the absolute content of chromophores, but also by their distribution in the skin [1]. Recent studies show that skin pigmentation affects the accuracy of non-invasive blood oxygen saturation measurements. For individuals with darker skin tones, pulse oximeters tend to provide falsely elevated SpO_2_ readings, particularly in hypoxic conditions [2]. Consequently, there is a need for new methods of assessing skin chromophore content, especially in relation to laser procedures. Skin color is a key diagnostic factor in the vast majority of dermatoses. In addition, by assessing the dynamics of color changes and their intensity, it is possible to determine whether the patient responds to the selected therapy [3]. The assessment of vascular lesion severity is conducted using several standardized qualitative scales, including the Investigator’s Global Assessment (IGA), where medical professionals evaluate the condition, and the Patient’s Global Assessment (PGA), where patients rate their symptoms.

Despite the ability of the human eye to discriminate between many colors (especially in detecting subtle changes in contrast), humans cannot precisely quantify the perception of a given color without the use of specialized instrumental tools. Therefore, physical examination cannot be the crucial element of skin diagnostics, including vascular skin, but should only be used to select appropriate diagnostic tests [4,5,6].

At the same time, the development of Optical Frequency Domain Reflectometry (OFDR) technology opens new possibilities in skin diagnostics. OFDR, based on Rayleigh scattering, enables precise measurements of parameters such as temperature, strain, and vibration [7]. This technique may be applied to the assessment of vascular skin changes, offering more accurate and objective results than traditional visual evaluation.

Vascular lesions, especially telangiectasia and erythema, are among the most common reasons why patients visit aesthetic medicine clinics. A wide range of services related to the removal of vascular lesions is currently available [8]. To remove telangiectasia by photothermolysis, high-energy lasers are used, causing selective overheating of tissues (the phenomenon of selective photothermolysis). During the procedure, the dilated vessel is irradiated with laser light, which is absorbed by the chromophore (hemoglobin) contained in the blood vessel [9]. The effect is a local increase in temperature to 70–72 °C, which causes denaturation of proteins and destruction of the vessel.

Studies by Dwiyan et al. confirm the effectiveness of Pulsed Dye Laser (PDL) in treating tufted angiomas. PDL demonstrates the ability to selectively damage blood vessels while minimizing injury to the surrounding skin, resulting in reduced redness and pain [10].

The IPL method (intense pulsed light) is a variation in photothermolysis, which uses diffused light with a wide band, emitted by a flash reflector. Unlike a laser, it is not a beam of coherent, polarized light with the same wavelength. IPL produces a wide spectrum of incoherent light waves which, due to their different lengths, can be absorbed by different chromophores. The head of the apparatus is placed on the skin, and the emitted light causes the vessel to close. This method is used for shallow telangiectasias [11]. Effective IPL therapy requires a series of treatments over several months.

In recent years, new methods for treating vascular skin lesions have been developed using advanced laser technologies. One example is the combination of alexandrite and Nd:YAG lasers, which has proven effective in treating cutaneous manifestations of Rendu–Osler disease [12].

Studies using the VISIA skin analysis system have shown that 532 nm wavelength laser therapy is effective in reducing facial vascular lesions in both women and men [13]. Interestingly, treatment effectiveness was not significantly dependent on skin phototype, patient age, or number of laser sessions, suggesting broad applications for this method in clinical practice. Additionally, the lack of correlation with key physiological factors indicates that research in this direction may yield many new insights.

Photodynamic therapy utilizing 5-aminolevulinic acid (ALA-PDT) represents an alternative treatment approach for rosacea. ALA penetrates pilosebaceous units where it converts to protoporphyrin XI, a chromophore that absorbs red light. This absorption triggers the generation of reactive oxygen species (ROS), which modulate inflammation, stimulate immune response, and inhibit excessive growth of pathological skin flora, including Demodex species [14,15,16,17,18,19].

Yang et al. [14] demonstrated that ALA-PDT therapy shows comparable efficacy to minocycline in treating moderate to severe rosacea. Research by Sun et al. [15] reported significant reductions in papules and overall skin improvement exceeding 67%, with marked reduction in erythema intensity observed one month post-treatment. This improvement may be attributed to ALA-PDT’s robust inflammatory modulation. However, given the intense inflammatory response generated during PDT therapy, its application for erythema reduction remains controversial [20].

Bao et al. [21] investigated a combination therapy incorporating three ALA-PDT sessions with three IPL treatments. Their findings demonstrated significant improvement in patient skin condition, including reduction in papular lesions, telangiectasia, and erythema. The researchers suggest that combining ALA-PDT therapy with subsequent IPL treatments may enhance PDT’s effectiveness in reducing erythematous manifestations.

The effectiveness of laser treatments and other treatments based on polychromatic light (IPL) depends primarily on the technical parameters of the device, the individual characteristics of the patient’s skin, the appropriate treatment technique, and, to a significant extent, on the optimal adjustment of the treatment parameters to clinical indications. It should be emphasized that there are currently no methods that enable a quantitative assessment of the effectiveness of laser treatments of vascular lesions. Therefore, it is not possible to precisely optimize the parameters of the treatment in relation to the individual characteristics of the patient’s skin, nor it is possible to quantitatively compare the effectiveness of treatments depending on the lasers or treatment techniques. Previously used methods of analyzing the response of vessels to high-energy radiation are based on subjective qualitative scales (assessment of the effects of the procedure by the doctor, patient satisfaction survey) or on semi-quantitative scales (e.g., rosacea severity score, erythema index).

Recent studies on laser treatments focus on the need to develop objective methods for evaluating the effectiveness of laser procedures in treating vascular skin lesions. The use of advanced image analysis systems may contribute to standardizing treatment outcome assessment and optimizing procedure parameters [13].

The aim of the study was to develop objective measurement methods that would allow for a quantitative assessment of the response of dilated blood vessels within the skin to treatments using high-energy light. The development of a methodology for quantitative measurements of the response of vascular lesions to high-energy radiation may contribute to the objectification of both the assessment of treatment effectiveness of laser procedures in aesthetic medicine, and the objective assessment of therapies used in dermatology and phlebology.

In light of recent research, this objective takes on particular significance. The development of objective measurement methods may not only improve the effectiveness of aesthetic procedures but may also contribute to a better understanding of how skin pigmentation affects medical diagnostics, which is crucial given the discoveries about the impact of skin color on skin perfusion [2].

## 2. Materials and Methods

### 2.1. Study Participants

The preliminary study involved 38 volunteers aged 20 to 61 who suffered from erythematous lesions, couperose skin, and/or rosacea. The research was performed following participant signatures on informed consent documents, comprehensive explanation of study objectives and protocol, and ethical board approval No. PCN/0022/KB1/11/I/20 of 19 May 2020. They were provided with post-treatment recommendations covering the principles of proper skin care after the treatments. The inclusion criteria were voluntary participation and presence of skin lesions qualifying for high-energy light treatments (vascular lesions such as erythema and/or telangiectasia on the face, discoloration on the skin of the hands and face—qualification conducted by a dermatologist). The exclusion criteria were age below 18 years, susceptibility to keloids and scar hypertrophy, implanted pacemaker or defibrillator, untreated diabetes, fresh tan, viral, bacterial, and fungal diseases of the skin, use of drugs or herbs with photosensitizing properties, pregnancy and lactation, cancer, taking anticoagulants, vitiligo, tattoos, permanent makeup in the treatment area, superficially applied dermal volumizers within the past half-year, neurotoxin injections during last 2 weeks, surgical interventions in the area of interest during the past 3 months, and no/withdrawal of consent to participate in the research.

### 2.2. Study Design

To quantify the skin’s response to high-energy light, a series of tests were performed on each volunteer using the following methods and techniques:

- Skin examination using a mexameter by Mexameter^®^ Courage-Khazaka Electronic (Germany).
- Hyperspectral imaging using a Specim IQ (Finland) hyperspectral camera with a spectral range of 400–1000 nm, together with a dedicated lighting system with flat spectral characteristics.
- Analysis of total reflectance using the hemispherical directional reflectometer from Solar 410, Surface Optics Corporation (USA), operating in the spectral range of 335–2500 nm.

The tests were carried out in each volunteer in the ROI (region of interest), which covered a 25 cm^2^ area on the cheek, determined arbitrarily based on the area with the greatest intensity of vascular lesions. ROI 1 represents the ROI before IPL procedures, and ROI 2 represents the area after a series of treatments.

#### 2.2.1. Mexametric Measurement

The Mexameter is a diagnostic device used to determine the content of melanin and hemoglobin in the skin. The mexameter probe is equipped with sixteen LEDs that emit light in three wavelengths: infrared (880 nm), red (660 nm), and green (568 nm). The receiver (photodetector) measures the light reflected by the skin. Since the amount of light emitted is precisely defined, it can quantify the light absorbed by the lesion, and thus determine the color intensity of the lesion—a darker lesion absorbs a greater amount of radiation. For hemoglobin measurement, the device determines the amount of absorbed and emitted light with a wavelength of 660 and 568 nm, while for two types of melanin, the wavelengths are 660 and 880 nm. In this study, the analyzed skin area was approximately 19.6 mm^2^ (disc diameter of approximately 5 mm) and was analyzed once [22,23,24,25,26]. Measurements were made in similar conditions of temperature, lighting, and air humidity. To ensure the accuracy and repeatability of the measurements, the same place on the patient’s skin (ROI) was precisely selected and tested during subsequent measurements. The applicator was applied to the examined area 6 times, and the average obtained for the 6 measurements was analyzed.

#### 2.2.2. Hyperspectral Imaging

Hyperspectral imaging merges two distinct non-invasive assessment methods: imaging and spectroscopy, enabling the determination of both the optical (image) and spectral (reflectance/absorption profile) characteristics of examined elements corresponding to individual pixels [27]. The spectral imaging device captures data through the detection of radiation energy with a specific energy intensity (I) in specific spatial coordinates (x, y) and wavelengths (λ). The Δx and Δy values assess the image’s spatial resolution, whereas the Δλ value determines the spectral resolution. To quantitatively identify the content and distribution of skin chromophores (mainly hemoglobin), the SPECIM IQ hyperspectral camera, SPECIM, Finland, was used, which allowed for the acquisition of images in the spectral range of 400 to 1000 nm with a spectral resolution of 2.94 nm, a spatial resolution of 512 × 512, and a physical pixel size of 17.58 × 17.58 μm.

The recording device, equipped with a 21 mm lens, was secured on a support stand at approximately 75 cm from the study subject. Volunteers were measured in a 90° seated position. Documentation included a reference reflectance standard, employing an enhanced lighting arrangement of two thermal sources providing consistent spectral distribution across the 400–1000 nm range. Each individual exposure required 14–18 ms under the specified illumination conditions, resulting in a complete spectral dataset acquisition of 204 frames completed in under 4 s. The image acquisition time (indirectly, the intensity of light) held significance since data acquisition lasting beyond 5 s resulted in artifacts related to the volunteers’ micromovements. The acquisition of the tested images was carried out in parallel with the acquisition of the reference panel (the calibration panel, also known as the gray panel), which allowed the determination of the absolute reflectance of materials in the tested wavelength range of 400–1000 nm. The projection was performed twice: before and after a series of treatments using high-energy light. Initial data preservation occurred in *.dat format, then transformed to the *.mat format for subsequent evaluation in the MATLAB environment. Based on this, the dependence of reflectance on wavelengths in the operating range of the hyperspectral camera (400–1000 nm) was determined for all tested materials.

#### 2.2.3. Hemispheric Directional Reflectance (HDR)

To determine the skin reflectance—before and after the IPL procedure—the total reflectance parameter was established. The SOC 410 Solar DHR reflectometer (Surface Optics Corporation from San Diego, CA, USA) was implemented. The equipment contains an integrated DHR (Directional Hemispherical Reflectance) analysis head, which enables the measurement of integrated reflectance from the surface—an angle of 20° for seven discrete spectral bands in the range of 335 nm–2500 nm: 335–380 nm, 400–540 nm, 480–600 nm, 590–720 nm, 700–1100 nm, 1000–1700 nm, and 1700–2500 nm. Directional reflectance was tested for 7 discrete spectral ranges for each volunteer. The measurement involved applying the reflectometer device to the volunteer’s skin in the ROI. Each ROI was sampled 6 times, and then the average reflectance for a given ROI was determined. The large diameter of the reflectometer’s measurement area (1.27 mm) ensured that the analysis concerned a large ROI, thus minimizing the measurement error caused by local differences in the content of skin chromophores. To determine the absolute reflection for the tested areas, two calibration coupons accredited by the American NIST (National Institute of Standards and Technology) were implemented. The reflectance was determined according to standard procedures.

### 2.3. The Erythema Reduction Treatments

Treatments for reducing erythema were performed using the Lumecca device, (InModeMD Ltd., Yokneam, Israel). It uses polychromatic light, commonly known as IPL (intense pulsed light). Lumecca emits noncoherent light with 515 nm–1200 nm and has a peak power of 9.9 kW for a spot size of 3 cm^2^. Lumecca has two modes of pulse duration; short (pulse duration ranges from 3 to 4 ms depending on the applied energy density) and long (pulse duration ranges from 5 to 6 ms depending on the applied energy density). Each of 38 volunteers received 3 treatments using polychromatic light. The treatment parameters were selected individually, based on the reaction of the skin (blood vessels) after applying an impulse with a specific energy density (Table 1). Treatment began with skin cleansing, followed by the application of ultrasound gel, which has cooling properties and reduces the radiation reflection coefficient at the skin/air interface. The skin reaction to the physical factor was then assessed. In volunteers whose skin reaction after the first treatment of the entire surface was not intense (any intense redness, swelling, or milky color of the vascular area), the procedure was repeated at a lower energy density (the second pass). The first pass was made in short mode, the second in long mode. After the treatment, regenerating and sun protecting products with an SPF 50+ protection factor were applied. The intervals between treatments were 3 to 4 weeks, following supplier-specified guidelines.

### 2.4. Statistic Analysis

For statistical analysis, Excel and Statistica 13 were used. The normality of the results’ distribution was analyzed through Shapiro–Wilk statistical examination, while variance consistency was evaluated via Leven’s statistical method. For normal distribution of results, a T test for related samples was used to determine the effect of the treatment, while for non-normal distribution, pre- and post-IPL intervention measurements underwent comparison utilizing Wilcoxon signed-rank statistical analysis.

## 3. Results

### 3.1. Mexametric Analysis

**Figure 1:**
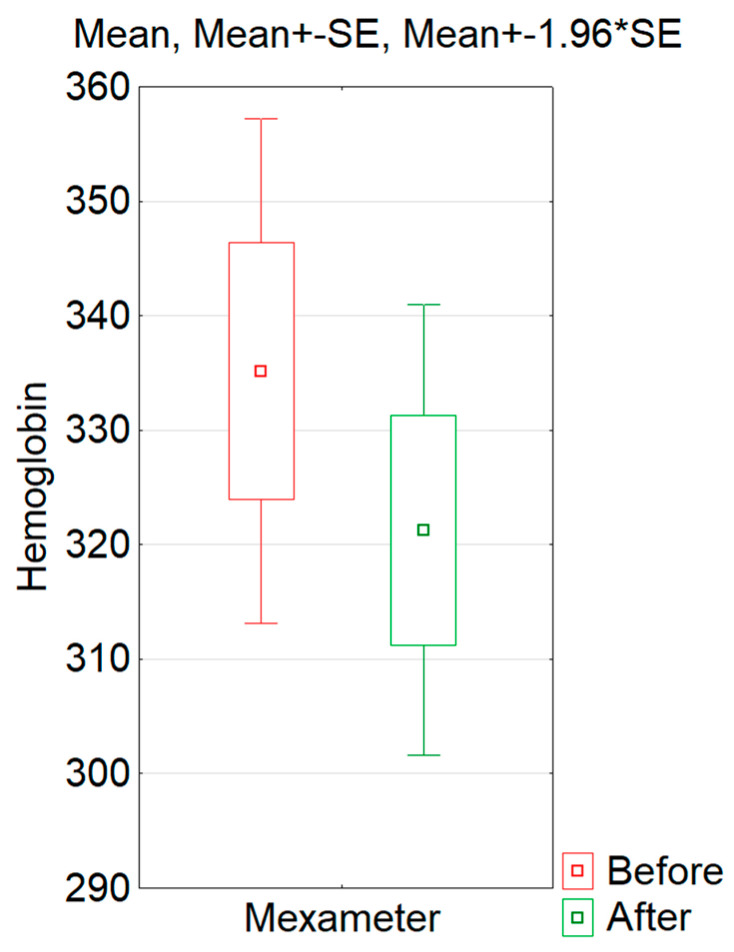
Values of hemoglobin scores of all volunteers in the ROI before and after the IPL procedure.

To quantify the hemoglobin content before and after a series of IPL treatments, the most popular method of measuring erythema—mexametry—was used. To determine the impact of IPL treatments on the intensity of erythema, hemoglobin content was measured in the ROI before and after therapy. The mexametric measurements had a normal distribution; therefore, the T test for related samples was used to determine the effect of the treatment. In the mexameter measurements, statistically non-significant differences were obtained in the hemoglobin content in the skin pre- and post-IPL treatments (t = 1.376; *p* = 0.177) (Figure 1). However, it was observed that after the procedures, the hemoglobin content decreased in 55% of volunteers. The reduction in the average hemoglobin content in the entire study group was 13.9, which represented 6% of the initial value.

### 3.2. Hyperspectral Analysis

**Figure 2:**
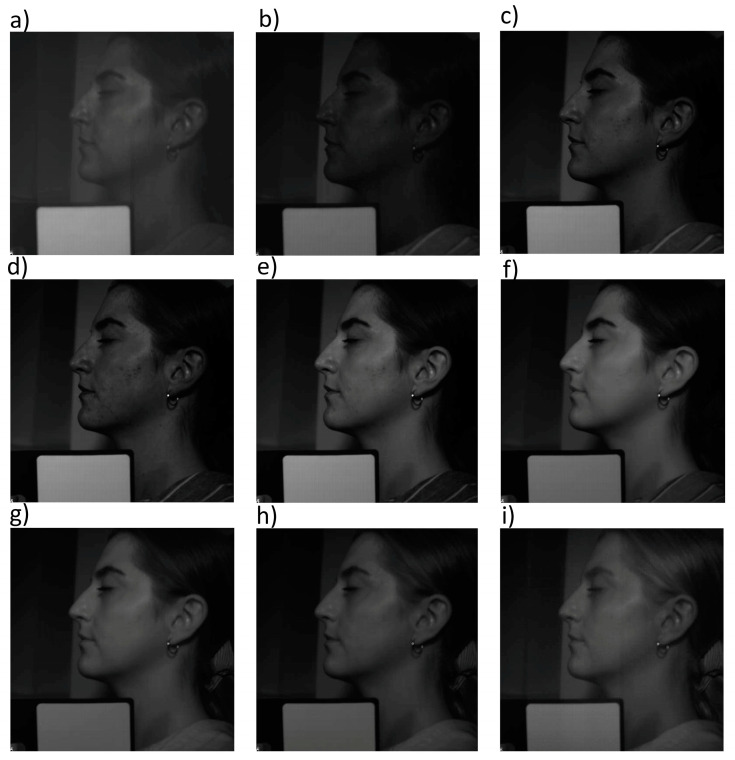
Hyperspectral images of volunteer #5 before the IPL procedure. Images for wavelengths 400 nm (**a**), 420 nm (**b**), 500 nm (**c**), 580 nm (**d**), 600 nm (**e**) 700 nm (**f**), 800 nm (**g**), 900 nm (**h**), and 1000 nm (**i**).

**Figure 3:**
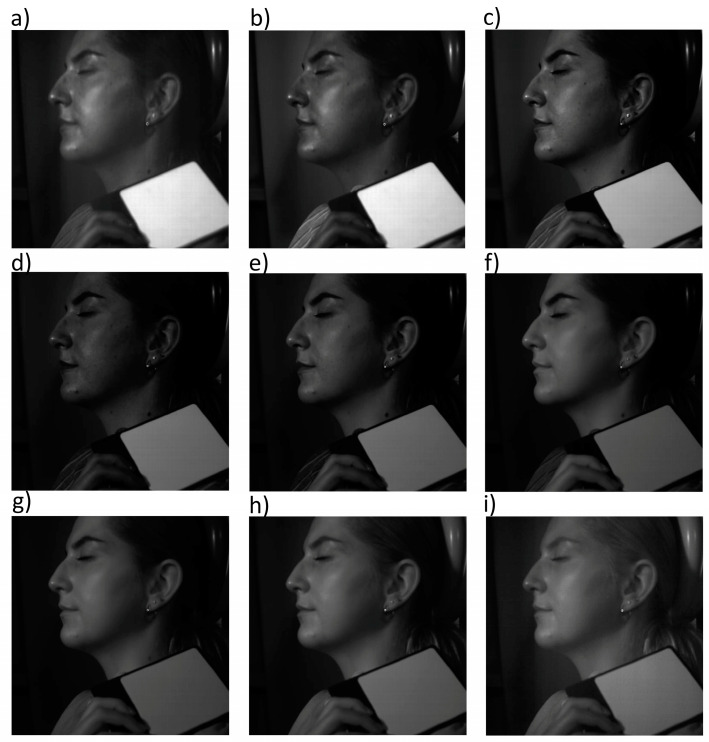
Hyperspectral images of volunteer #5 after the IPL procedure. Images for wavelengths 400 nm (**a**), 420 nm (**b**), 500 nm (**c**), 580 nm (**d**), 600 nm (**e**) 700 nm (**f**), 800 nm (**g**), 900 nm (**h**), and 1000 nm (**i**).

Figure 2 and Figure 3 show example images of hyperspectral analysis for volunteer #5 before and after the IPL procedure, respectively. Hyperspectral images were recorded in the spectral range of 400–1000 nm with a resolution of 2.94 nm. These figures show images for representative wavelengths: 400 nm, 420 nm, 500 nm, 580 nm, 600 nm, 700 nm, 800 nm, 900 nm, and 1000 nm. The images also show the calibration panel, which enabled the determination of total reflectance for the ROIs of the volunteers’ skin.

**Figure 4:**
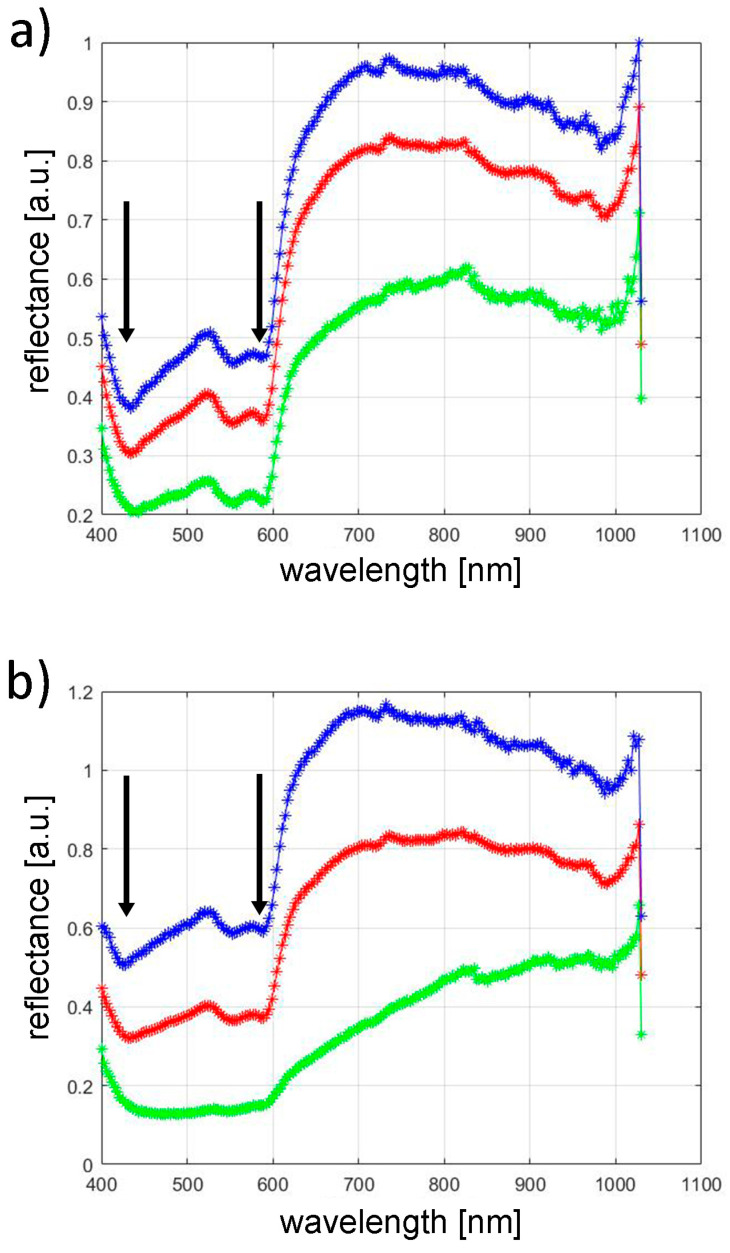
Hyperspectral profiles for volunteer’s #5 ROI before (**a**) and after (**b**) the IPL procedure. The arrows mark the wavelengths of 420 nm and 580 nm, for which there is a maximum absorption of oxyhemoglobin. Blue line—maximum reflectance in the ROI, red line—mean reflectance in the ROI, green line—minimum reflectance in the ROI.

Hyperspectral profiles of the ROIs were determined from the obtained hyperspectral images for all the volunteers before and after the IPL procedure. Figure 4 shows exemplary hyperspectral profiles for volunteer #5. The blue graph represents the maximum. Within the visualization, blue curves indicate the highest ROI reflective properties, red lines show mean values, and green traces represent minimal readings. Converting the spectral information to grayscale levels via standardized algorithms allows pixel intensity to directly correspond with total reflectance. This enables the systematic identification of both the brightest and darkest elements in the examined region. Mean reflectance values are calculated by totaling ROI pixel intensities and dividing this value by the number of measured points.

The investigation extracted reflectance values corresponding to primary oxyhemoglobin absorption wavelengths (420 nm and 580 nm) from spectral profiles. These measurements, gathered from participant ROIs, underwent pre–post-IPL comparison. Shapiro–Wilk analysis indicated non-normality in data distribution. Statistical comparison of pre- and post-treatment measurements at 420 nm and 580 nm utilized Wilcoxon signed-rank methodology, analyzing each wavelength separately.

**Figure 5:**
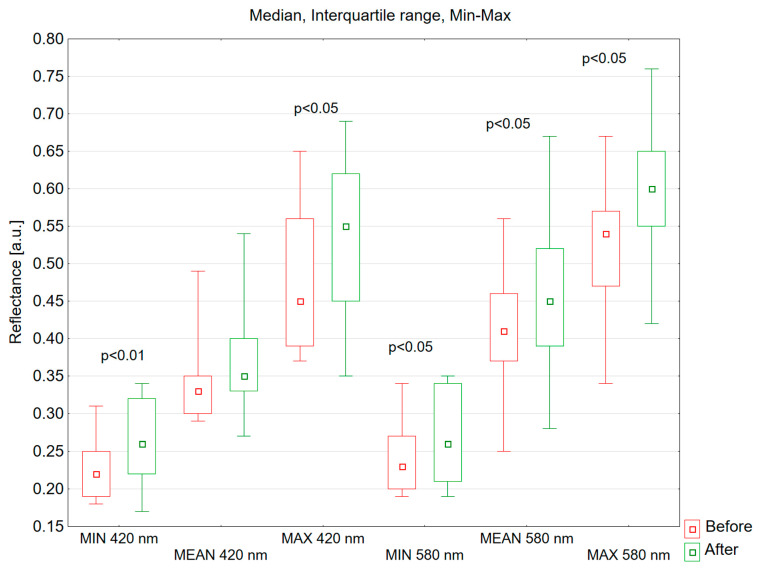
Mean, minimum, maximum reflectance values for the ROI of all tested volunteers at 420 nm and 580 nm before and after the IPL procedure. MIN—minimum reflectance value; MEAN—mean reflectance value; MAX—maximum reflectance value.

For the 420 nm spectrum, the median of mean reflectance values shifted from 0.330 pre-intervention to 0.350 following treatment. This reflectance enhancement lacked statistical significance (*p* = 0.272), as illustrated in Figure 5. At a wavelength of 580 nm, the increase in skin reflectance after the treatment was statistically significant (*p* = 0.016). The median of mean reflectance value in the study group increased from 0.410 to 0.450 following the treatment.

After a series of IPL treatments, there was a statistically significant increase in the minimum reflectance value at both 420 nm and 580 nm wavelengths (420 nm, *p* = 0.005; 580 nm, *p* = 0.023) (Figure 5). At a wavelength of 420 nm, before the IPL treatment, the median of the minimum reflectance was 0.220, and after the treatment, it was 0.260. At a wavelength of 580 nm, the median value of the minimum reflectance before the treatment was 0.230, and after the treatment, it was 0.260.

After IPL treatments, the maximum skin reflectance values at 420 nm and 580 nm wavelengths showed statistically significantly increases compared to the skin reflectance before the treatments (420 nm, *p* = 0.031; 580 nm, *p* = 0.023) (Figure 5). At a wavelength of 420 nm, the median value of the maximum reflectance before the treatment was 0.450, and after the treatment, it was 0.550. At a wavelength of 580 nm, the median value of the maximum reflectance before the treatment was 0.540, and after the treatment, it was 0.600.

### 3.3. Hemispheric Directional Reflectance

Measurements were also made using a hemispheric directional reflectometer, which enabled the determination of reflectance for seven discrete bands within the spectrum: 335–380 nm, 400–540 nm, 480–600 nm, 590–720 nm, 700–1100 nm, 1000–1700 nm, and 1700–2500 nm. Measurements were made at a 20 degree angle.

**Figure 6:**
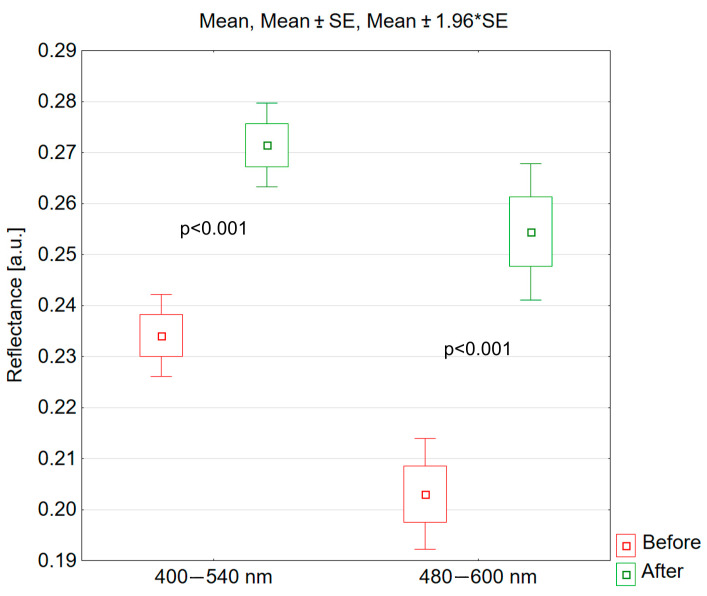
Reflectance of skin with erythema before and after IPL for the spectral ranges of 400–540 and 480–600 nm.

In the wavelength range of 400–540 nm, following the IPL treatment, the skin reflectance increased statistically significantly (t = −7.686; *p* < 0.001). In this spectral range, skin reflectance increased in 97% of volunteers; its average value increased by 0.037, which represents 16.0% of the value before the series of IPL treatments (Figure 6).

Similarly, after the IPL treatment, skin reflectance increased statistically significantly for the spectral range of 480–600 nm (t = −6.674; *p* < 0.001). The reflectance value increased in 89% of volunteers; the average reflectance value increased by 0.051, which represents 25.3% of the reflectance value before the procedure (Figure 6).

The use of IPL treatments on skin with erythema had a statistically non-significant effect on its reflectance in the spectral ranges of 335–380 nm, 590–720 nm, 700–1100 nm, 1000–1700 nm, and 1700–2500 nm.

## 4. Discussion

Facial erythema is one of the most common aesthetic problems that patients, especially those with Fitzpatrick I-III skin phototypes, report to dermatologists. The appropriate assessment of the cause and severity of facial erythema is necessary for the correct selection of therapy required to reduce redness. The first-line treatments of extensive vascular lesions on the face are methods based on high-energy light sources, such as lasers and IPL [28,29,30].

An analysis of the literature data shows that in most cases, the assessment of vascular changes—before and after therapy—is made using subjective qualitative scales [30,31,32]. From published works where scientists pursued quantitative analysis of vessel change, most used tools implemented image analysis and processing algorithms: for example, Visia by Canfiled (USA) [33,34], Antera 3D by Miravex (Ireland) [35,36], and Primos by GFMesstechnik GmbH (Germany) [37,38]. Initially, these systems utilize proprietary analytical algorithms that manufacturers keep confidential. This lack of transparency prevents independent verification of the methodology’s reliability, reproducibility, precision, and accuracy. An additional limitation lies in these tools’ generic analytical approach, lacking specialization for particular skin conditions, thus requiring broad-spectrum processing techniques that may not suit diverse dermatological cases. Furthermore, market accessibility demands dictate cost-effective and user-friendly designs, creating significant disparity when comparing these basic imaging tools with sophisticated scientific instruments like hyperspectral systems or perfusion analyzers. Significantly more precise and objective assessment of pathologically altered blood vessels can be achieved using optical coherence tomography (OCT). Liu et al. described the application of OCT as a tool for diagnosing the diameter and depth of abnormal blood vessels in port wine stain lesions [39]. Jill et al. [40] demonstrated the utility of OCT for imaging blood vessel patterns, their diameter, depth, and morphology in hemangiomas and port wine vascular malformations. Scientific analyses also employ three-dimensional photography to assess volume reduction in hypertrophic hemangiomas and vascular malformations [41]. The limitation of 3D photography lies in its application to flat lesions where no tissue volume changes occur.

In view of the above-mentioned limitations and methods of assessing vascular changes known from the literature, new techniques for quantitative, objective analysis of vascular changes before and after a series of IPL procedures have been proposed.

The studies conducted using the mexameter verified the low usefulness of this method in assessing the effectiveness of vascular lesion removal. The reduction in the average hemoglobin content in the entire study group was 13.9, which represented 6% of the initial value; however, these changes did not correspond to clinical data, which subjectively indicated a greater improvement. Mexametry is a quick, cheap, and simple method that can be used to determine the severity of vascular lesions, but its use is controversial when compared with the results of medical procedures. This may be due to the fact that the measuring surface of the mexameter has a relatively small area (from a few to 20 mm^2^). Consequently, a relatively small area of skin is sampled, which may not be representative of the entire anatomical area (e.g., cheek). Another problem is the inability to sample the skin in exactly the same place after a series of procedures (in this case, IPL). As a result, the reproducibility of results over a longer period of time may be unsatisfactory, which was confirmed in this research.

The evaluation of vascular modifications utilized spectral imaging technology. This study employed imaging equipment operating within 400–1000 nm wavelengths, corresponding to peak absorption/reflection patterns of cutaneous chromophores, particularly hemoglobin. Analysis revealed pre-IPL median reflectance measurements at 420 nm showing values of 0.330, and after the procedure, 0.350; this increase was statistically non-significant (*p* = 0.272). At a wavelength of 580 nm, the increase in skin reflectance after the treatment was statistically significant (*p* = 0.016). The median value of the average reflectance increased from 0.410 to 0.450 following the treatment. The median reflectance increased statistically significantly for the maximum and minimum values at both wavelengths, 420 nm and 580 nm. The hyperspectral imaging method used in this study has several advantages over other spectroscopic methods that are used to assess the severity of vascular lesions [42,43,44,45]. Traditional spectroscopic approaches examine minimal skin regions—under 5 mm^2^ in area. Similar to mexametric evaluation, such limited sampling may inadequately represent broader cutaneous zones, while precise location replication across treatment intervals poses significant challenges. Additionally, spectroscopic measurements utilize perpendicular beam orientation, restricting analysis to narrow angular parameters. In contrast, hyperspectral imaging enables reflection capture across multiple angles, defined by the camera lens’s field of view. Therefore, hyperspectral imaging may be considered a promising method for the quantitative assessment of the severity of skin vascular lesions.

Total reflectance measurements confirm the results obtained with hyperspectral imaging. A statistically significant increase in reflectance, caused by a decrease in hemoglobin content, was observed only for the spectral ranges of 400–540 nm and 480–600 nm. No significant differences were obtained for the other spectral ranges. Although the hemispheric directional reflectometer is not as precise an instrument as the hyperspectral camera—since it allows the analysis of reflectance in relation to spectral intervals, rather than single wavelengths like the hyperspectral camera—this method should also be considered very promising in the quantitative assessment of vascular changes. The advantages of the reflectometer measurement include the following: speed—measurement takes less than 5 s; simplicity—the operator simply needs to apply the aperture of the device tangentially to the skin and press the trigger to obtain an immediate result; and broad spectral range—measurement across wavelengths from 335 to 2500 nm. A similar diagnostic technique is reflectance spectroscopy in vivo, which uses fiber optic probes to transmit light of specific wavelength ranges onto the tissue surface and to measure the intensity of reflected light. The difference between delivered and reflected light can provide information about changes in erythema intensity and vascular changes in the skin. In their studies, Troilus and Ljunggren demonstrated that reflectance spectroscopy can be a more precise measurement technique than laser Doppler examination [46,47].

## 5. Conclusions

The study of the response of vascular lesions to high-energy IPL light, using mexametric measurements, hemispheric directional reflectance, and hyperspectral imaging, allowed the following conclusions to be drawn:

1. Mexametric measurements do not allow for a quantitative assessment of the response of skin blood vessels to high-energy light.
2. Hyperspectral imaging is an effective method for the quantitative assessment of vascular lesions.
3. The most effective assessment of vascular changes using hyperspectral imaging is obtained at wavelengths of 420 nm and 580 nm.
4. The results of measurements using hemispheric directional reflectance confirm the results obtained from hyperspectral imaging and allow for a quick, accurate, and repeatable assessment of vascular lesions.

## Figures and Tables

**Table 1:** Average energy densities used in volunteers during erythema reduction treatments using Lumecca device.

Volunteer	First Treatment		Second Treatment		Third Treatment	
	Energy Density (J/cm^2^)					
	First Pass	Second Pass	First Pass	Second Pass	First Pass	Second Pass
Mean	12.39	9.81	13.26	11.21	14.24	11.95
SD	0.96	0.39	0.71	0.69	0.74	0.82

## Data Availability

The datasets used and/or analyzed during the current study are available from the corresponding author on reasonable request.
